# Abdominal Cocoon Syndrome Masquerading as Intussusception on Computed Tomography: A Case Report

**DOI:** 10.7759/cureus.113001

**Published:** 2026-07-20

**Authors:** Suad Jamal, Sana Jamal, Mohammed Temeiza, Şükrü Çolak

**Affiliations:** 1 Department of Surgery, Washington University of Health and Science, Rawabi, PSE; 2 Department of General Surgery, Nişantaşı University, Istanbul, TUR

**Keywords:** abdominal cocoon syndrome, fibrocollagenous membrane, intussusception, laparoscopy, small bowel obstruction, target sign

## Abstract

Abdominal cocoon syndrome (ACS), also known as sclerosing encapsulating peritonitis (SEP), is a rare cause of mechanical small bowel obstruction characterized by partial or complete encasement of the small bowel within a fibrocollagenous membrane. Owing to its nonspecific clinical and radiological presentation, preoperative diagnosis remains challenging.

A 38-year-old woman with a history of four previous Caesarean sections presented with one week of progressive colicky abdominal pain, abdominal distension, and absolute constipation that worsened during the preceding 24 hours despite conservative management. She reported a five-year history of recurrent, self-limiting episodes of bowel obstruction beginning two to three months after her last Caesarean section. Contrast-enhanced computed tomography (CT) was initially interpreted as a 6-cm distal jejunojejunal intussusception with a characteristic target sign. Diagnostic laparoscopy, however, revealed partial encapsulation of the small bowel by a dense fibrocollagenous membrane, consistent with secondary type I ACS. Complete laparoscopic membrane excision and adhesiolysis were successfully performed. Retrospective postoperative review of the CT images demonstrated clustering of small bowel loops with partial fibrocollagenous membrane encasement that had been overlooked on the initial interpretation.

The postoperative course was uneventful. Oral intake was resumed on postoperative day 3, and the patient was discharged on postoperative day 5. Histopathological examination demonstrated fibrocollagenous tissue with chronic inflammatory infiltrates. After excluding other recognized secondary causes, the patient’s history of four previous Caesarean sections was considered the most likely predisposing factor. At two-year follow-up, the patient remained asymptomatic with no recurrence of bowel obstruction.

ACS may mimic intussusception on CT, creating an important diagnostic pitfall in patients with recurrent small bowel obstruction. This case highlights the importance of maintaining a high index of clinical suspicion and demonstrates the short-term technical feasibility of laparoscopic diagnosis and management in carefully selected patients when performed by experienced surgeons.

## Introduction

Abdominal cocoon syndrome (ACS), first described by Foo et al. in 1978 [1], is a rare clinical entity characterized by partial or complete encapsulation of the small bowel by a fibrocollagenous membrane, leading to intestinal obstruction. It is also referred to as sclerosing encapsulating peritonitis (SEP) or peritonitis chronica fibrosa incapsulata [2].

ACS may be classified as primary (idiopathic) or secondary, the latter associated with conditions such as previous abdominal surgery, chronic ambulatory peritoneal dialysis, abdominal tuberculosis, β-blocker use, and malignancy [3]. Due to its rarity and nonspecific clinical and radiological presentation, ACS is rarely diagnosed preoperatively and is often discovered during surgical exploration [4].

We report a case of secondary type I ACS in a patient with a history of four previous Caesarean sections who was initially misdiagnosed with jejunojejunal intussusception on contrast-enhanced CT. This case highlights an uncommon radiological pitfall and supports the short-term technical feasibility of laparoscopic diagnosis and management in carefully selected patients.

## Case presentation

A 38-year-old woman (gravida 4, para 4) presented with a one-week history of colicky abdominal pain, progressive abdominal distension, and absolute constipation, which had worsened during the preceding 24 hours. She reported a five-year history of recurrent, spontaneously resolving episodes of abdominal pain, distension, and bowel obstruction occurring approximately three to four episodes annually, each managed conservatively with intravenous fluids and bowel rest in peripheral clinics. Because of the recurrent nature of her symptoms, a colonoscopy had been performed two years earlier to exclude underlying colonic pathology and was unremarkable.

Her past surgical history was significant for four previous Caesarean sections, the most recent performed five years before presentation. She had no significant past medical history and was not taking any regular medications.

On examination, the patient was afebrile (37.1°C) and hemodynamically stable. Abdominal examination revealed distension and diffuse tenderness on deep palpation without signs of peritonitis or palpable masses. Bowel sounds were hyperactive on auscultation. Digital rectal examination was unremarkable. Laboratory investigations, including white blood cell count (8.5 × 10³/µL) and C-reactive protein (<5 mg/L), were within normal limits. Her serum electrolytes, renal function tests, and lactate levels were also unremarkable.

Because the patient remained hemodynamically stable and showed no signs of bowel ischemia or peritonitis, an initial trial of conservative management was considered appropriate.

She was admitted for bowel rest, intravenous fluids, and close clinical observation. However, after one week of conservative management, her symptoms failed to improve, with progressive worsening of abdominal pain during the final 24 hours of hospitalization.

**Figure 1:**
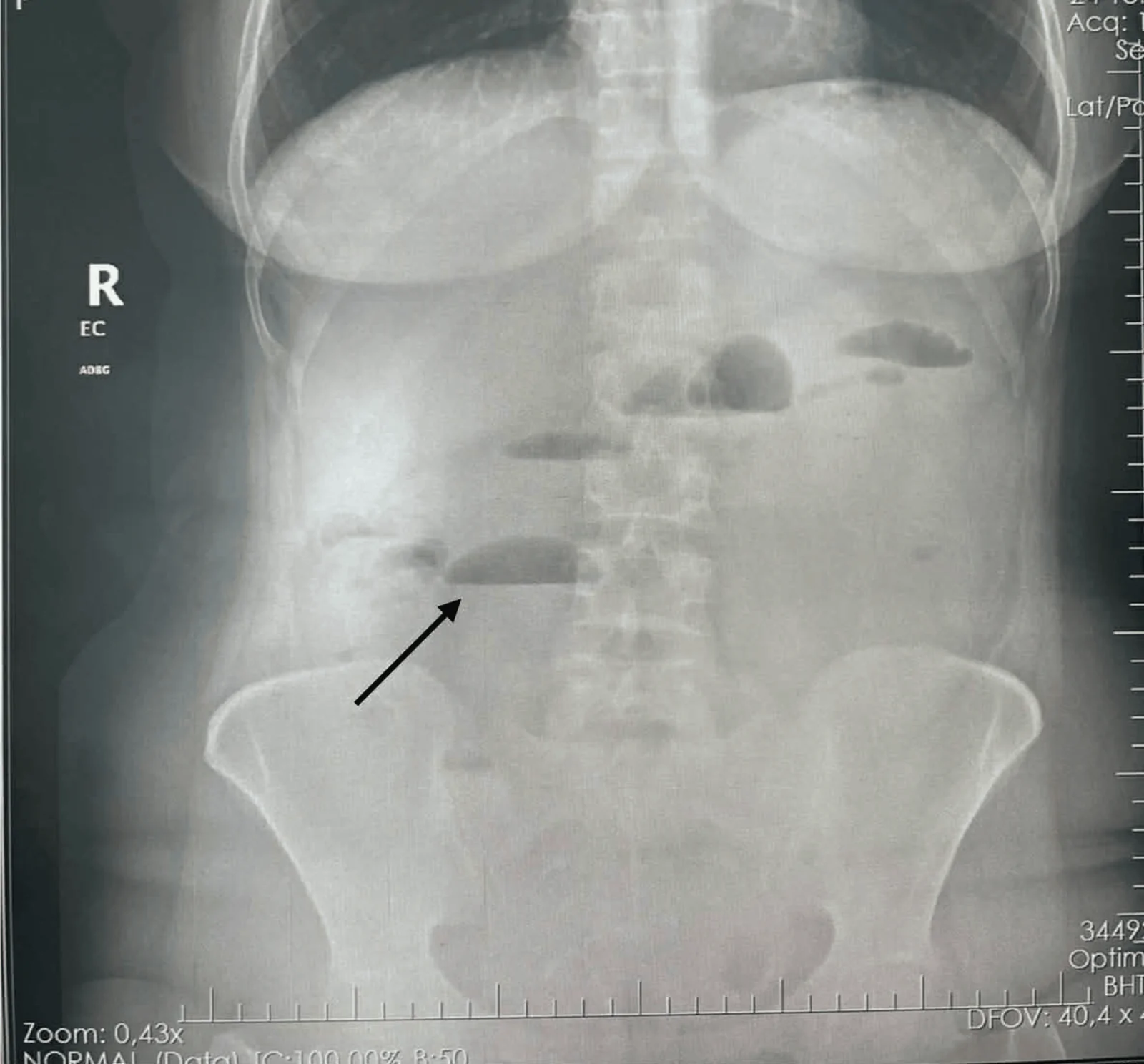
Plain Abdominal Radiograph The image reveals multiple air-fluid levels (arrow), which are classic radiographic findings confirming small bowel obstruction.

An erect abdominal radiograph demonstrated multiple centrally located dilated small bowel loops with several air-fluid levels, consistent with mechanical small bowel obstruction (Figure 1). Given these findings, contrast-enhanced CT of the abdomen and pelvis was subsequently performed for further evaluation of the cause of obstruction.

**Figure 2:**
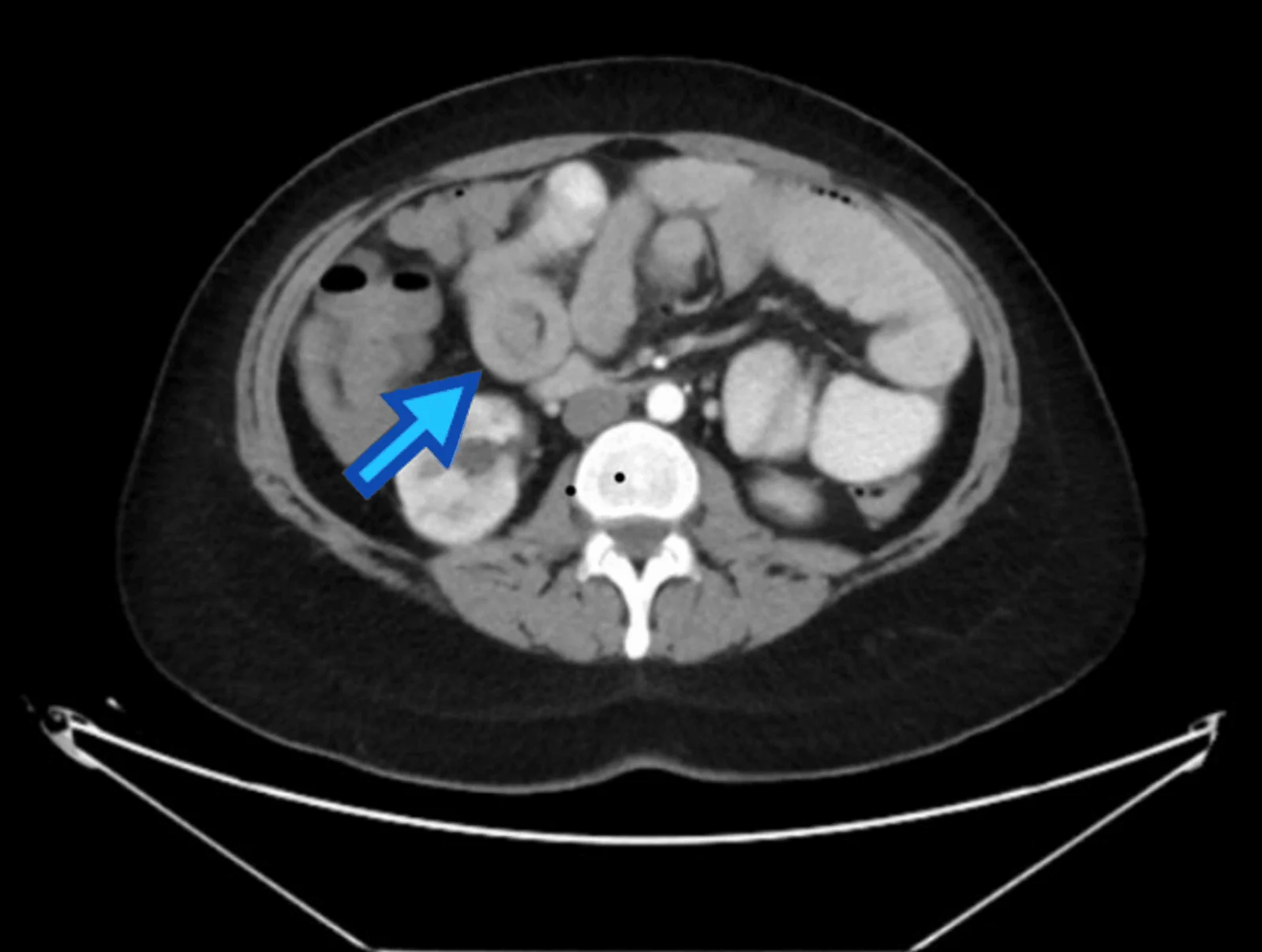
Axial Contrast-Enhanced CT of the Abdomen An axial view from the contrast-enhanced CT scan demonstrates the “target sign” or “bowel-within-bowel” configuration (arrow) in the distal jejunum. CT: computed tomography

**Figure 3:**
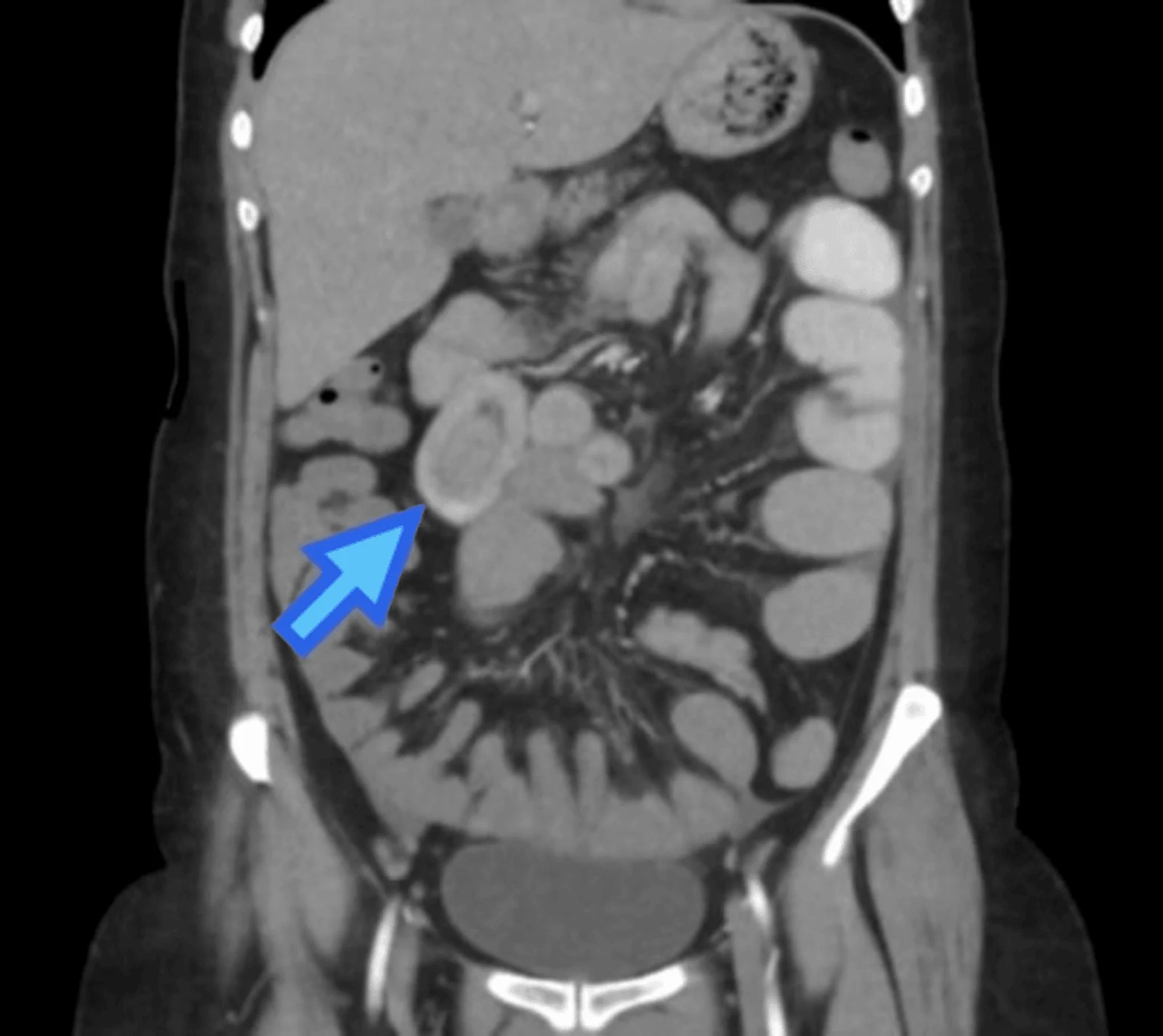
Coronal Contrast-Enhanced CT of the Abdomen A coronal view from the contrast-enhanced CT scan demonstrates the “target sign” or telescoping appearance (arrow) in the distal jejunum. CT: computed tomography

The initial contrast-enhanced CT examination demonstrated a short-segment jejunojejunal intussusception in the distal jejunum, measuring approximately 6 cm in length. The intussusception exhibited the characteristic target (bowel-within-bowel) configuration on axial images and an elongated telescoping appearance on coronal reformatted images. Associated proximal jejunal dilatation, with a maximum bowel diameter of 36 mm, and multiple air-fluid levels were present, consistent with mechanical small bowel obstruction, without any identifiable mass or lead point (Figures 2, 3).

**Figure 4:**
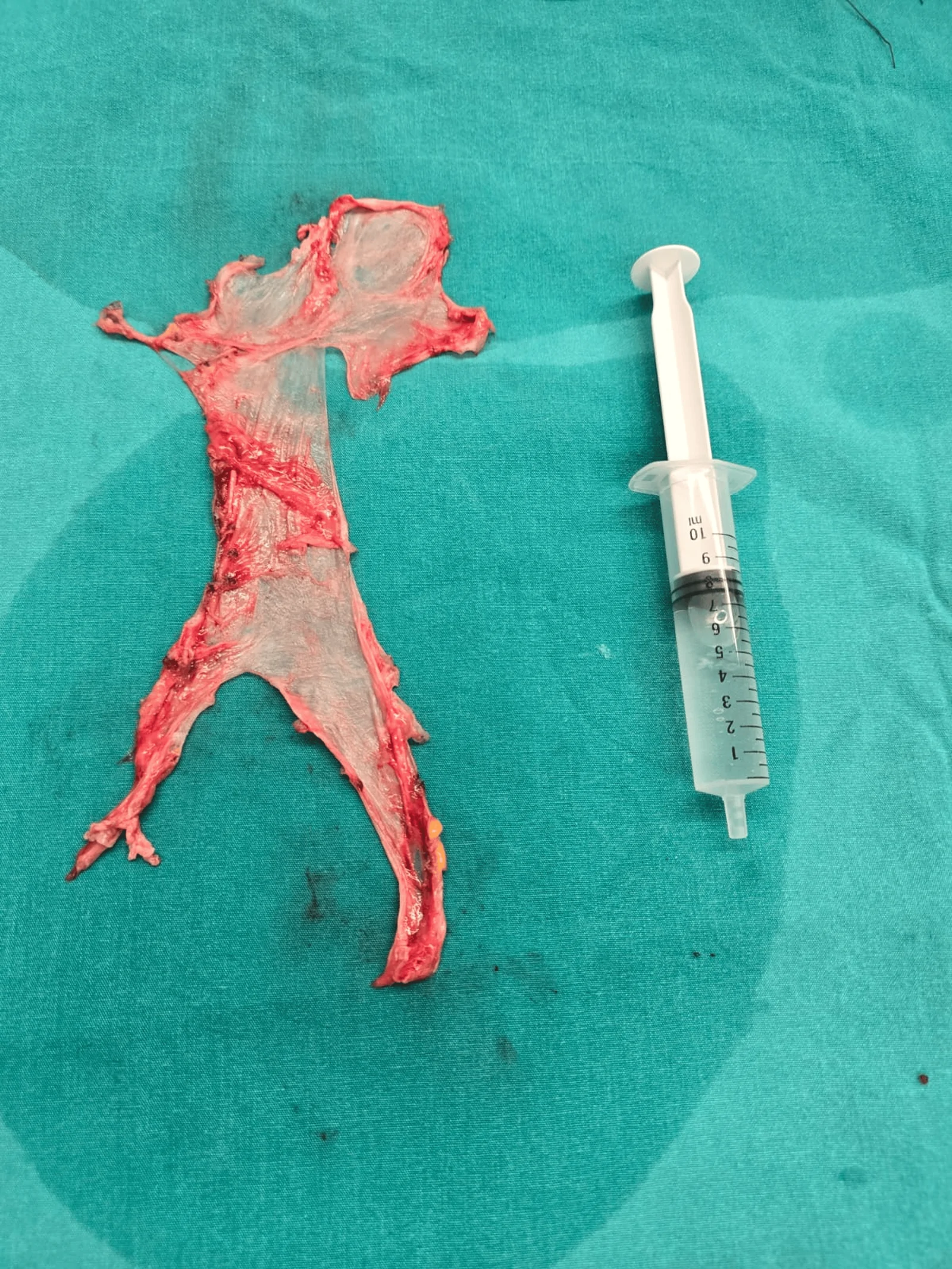
Gross Macroscopic Appearance of the Excised Encapsulating Membrane The image demonstrates the thick, vascular fibrocollagenous membrane immediately following complete laparoscopic excision. A syringe is included for dimensional reference.

Based on these findings, the patient underwent urgent diagnostic laparoscopy. Intraoperatively, no intussusception was identified. Instead, a segment of small bowel was partially encased within a thick, whitish, fibrous membrane, consistent with secondary type I abdominal cocoon syndrome (Figure 4).

**Figure 5:**
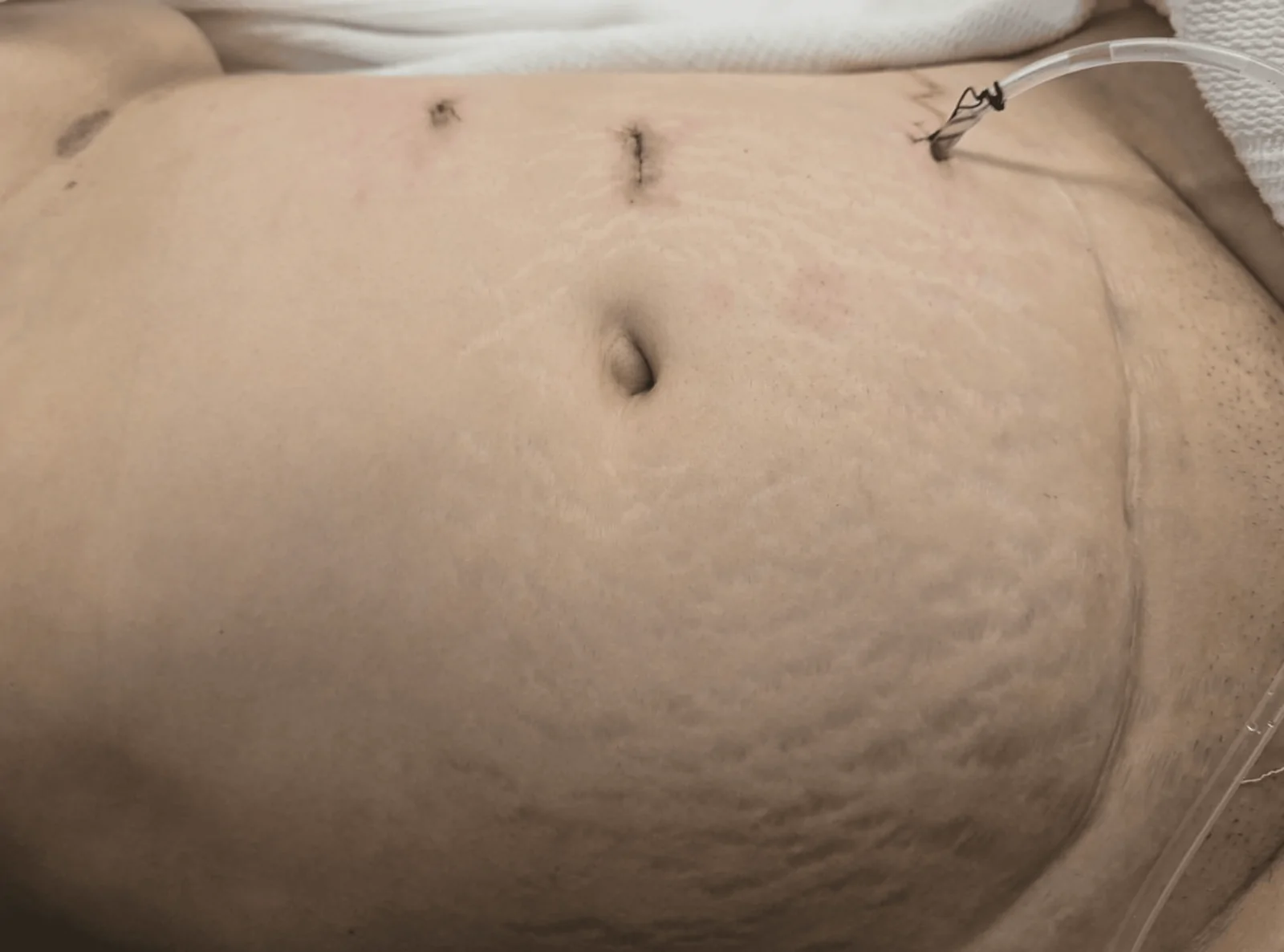
Post-laparoscopic Abdominal View With Port Incisions Postoperative abdominal view demonstrating laparoscopic port-site incisions following successful laparoscopic excision of the fibrocollagenous membrane.

Careful laparoscopic sharp dissection was performed to excise the membrane completely and release the entrapped bowel loops. Adhesiolysis was done, and the entire small bowel was carefully released from the membrane with blunt and fine dissection using endoscopic scissors. No bowel ischemia or serosal injury was identified. No intraoperative evidence of malignancy, endometriosis, or other secondary causes of sclerosing encapsulating peritonitis was identified. The procedure was technically demanding due to the membrane’s density and vascularity but was completed successfully without conversion to laparotomy (Figure 5).

**Figure 6:**
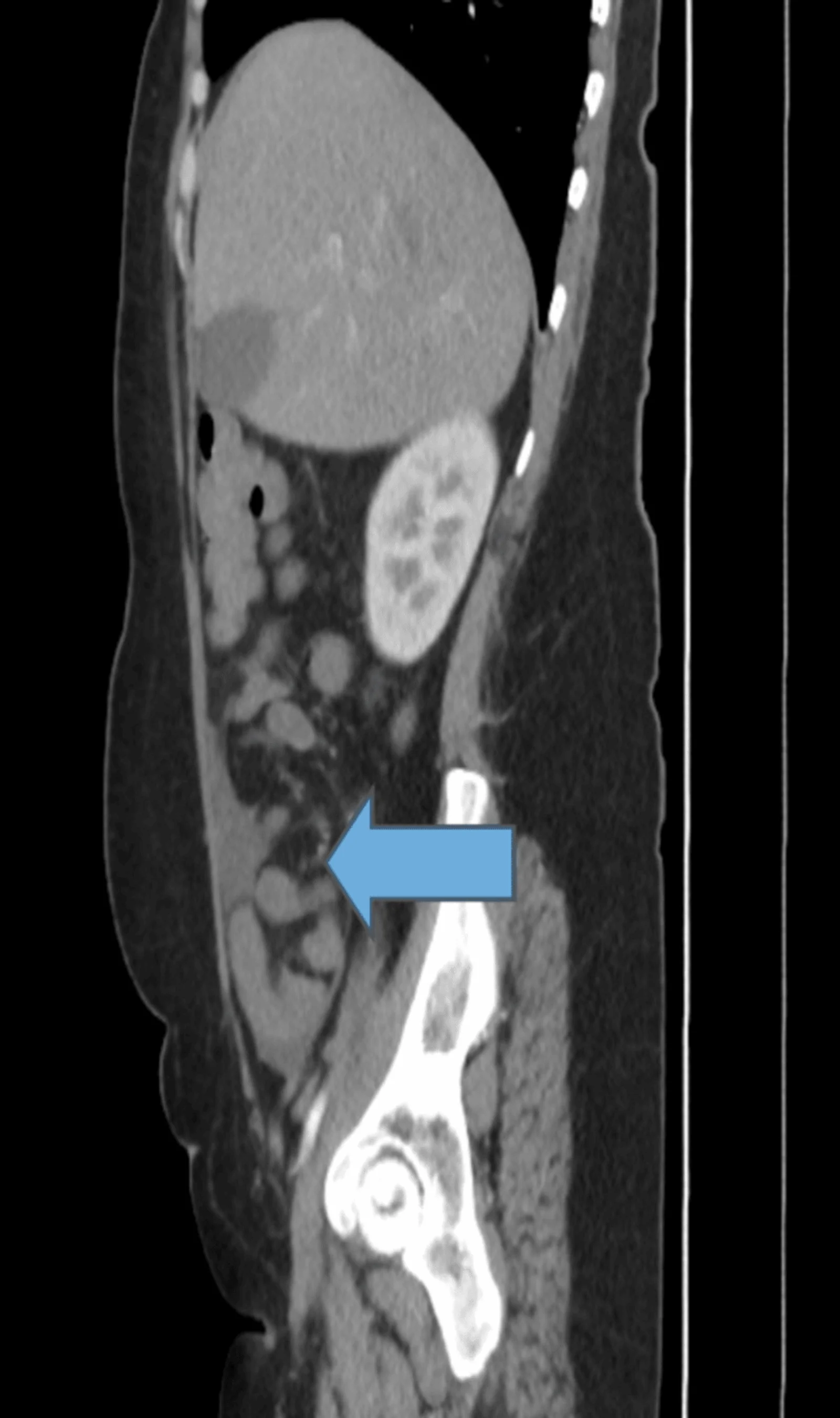
Sagittal Contrast-Enhanced Abdominal CT Image Sagittal contrast-enhanced CT view demonstrating the posterior demarcation of the cocoon sac (arrow). A small amount of intraperitoneal fluid was also identified. CT: computed tomography

**Figure 7:**
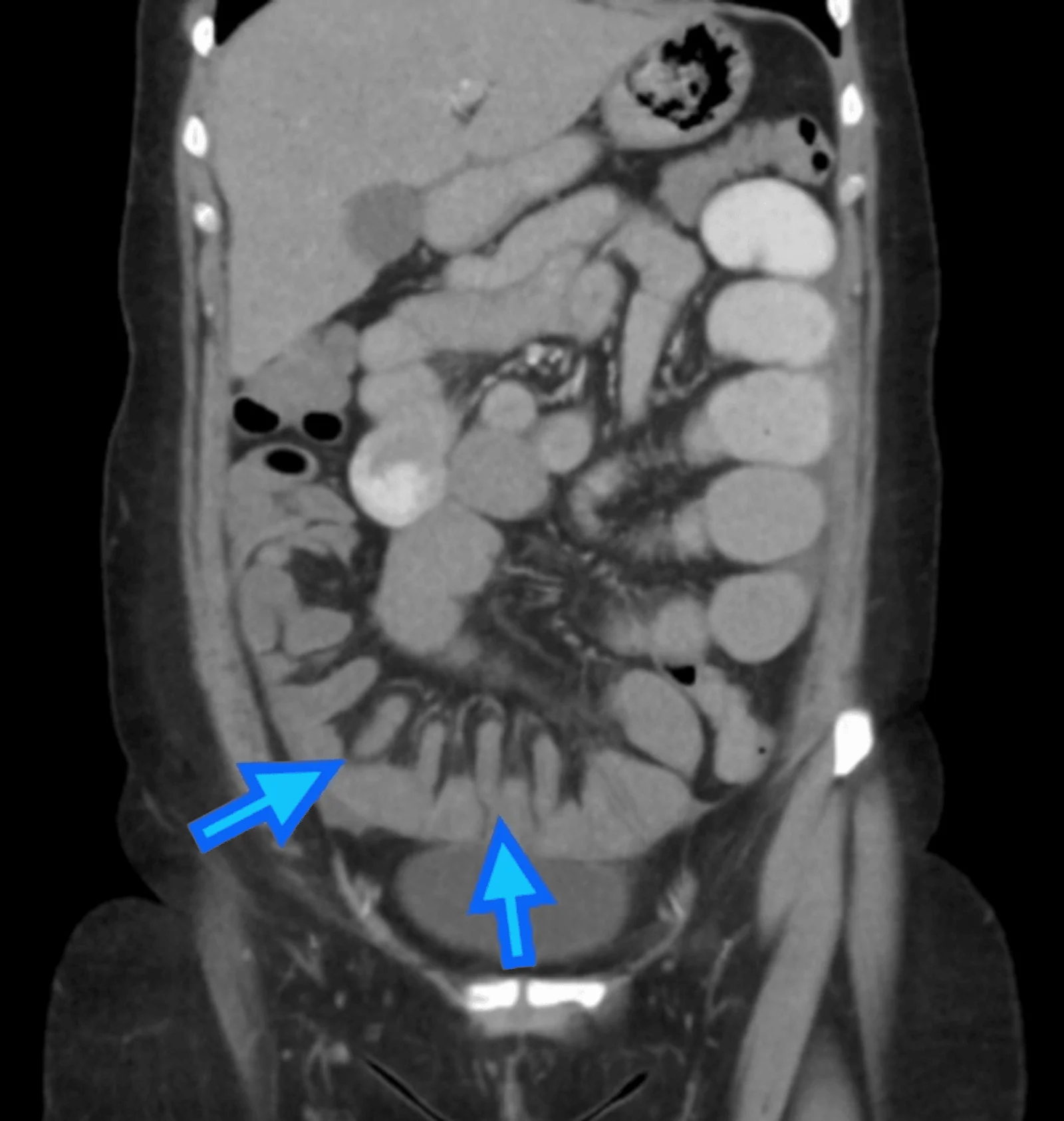
Coronal Contrast-Enhanced Abdominal CT Image A coronal view from the contrast-enhanced CT image demonstrating the verticalized, crowded, and U-shaped configuration of the small bowel loops trapped within the central abdomen, in a typical cocoon pattern (arrow), producing the characteristic “cauliflower sign.” CT: computed tomography

**Figure 8:**
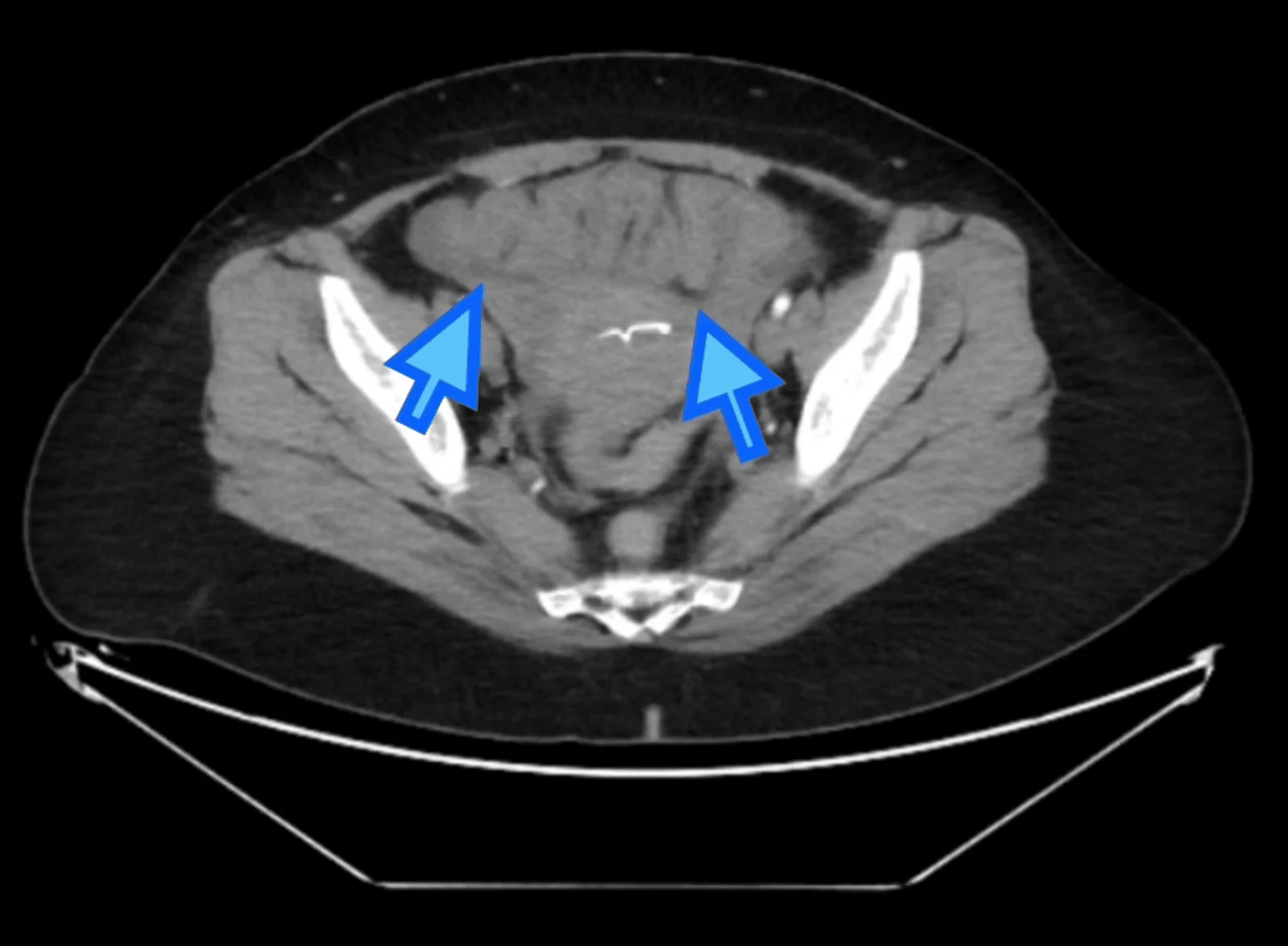
Axial Contrast-Enhanced CT Axial contrast-enhanced CT image illustrating a dense, centralized aggregation of multiple small bowel loops adjacent to the uterine fundus. The loops are tightly clustered and encapsulated within a contrast-enhancing membrane (arrows). CT: computed tomography

Retrospective postoperative review of the contrast-enhanced CT images (axial, coronal, and sagittal reformatted views) demonstrated imaging findings compatible with ACS. Clustered small bowel loops were enclosed within an enhancing membrane, best appreciated on the sagittal reformatted images (Figure 6). The encapsulated bowel loops appeared crowded, vertically oriented and conﬁned to the central abdomen, producing a characteristic cauliflower configuration on the coronal and axial images (Figures 7, 8). Mild proximal small bowel dilatation, most pronounced in the jejunum (maximum diameter: 36 mm), with multiple air-fluid levels, was also present, consistent with mechanical small bowel obstruction. A small amount of intraperitoneal fluid was also identified. The characteristic CT findings of ACS had been overlooked on the initial examination because they were overshadowed by the more conspicuous target appearance of the intussusception.

The postoperative course was uneventful. Liquid diet was resumed on postoperative day 1, which was progressed to a soft diet by postoperative day 3, and the patient was discharged home on postoperative day 5.

**Figure 9:**
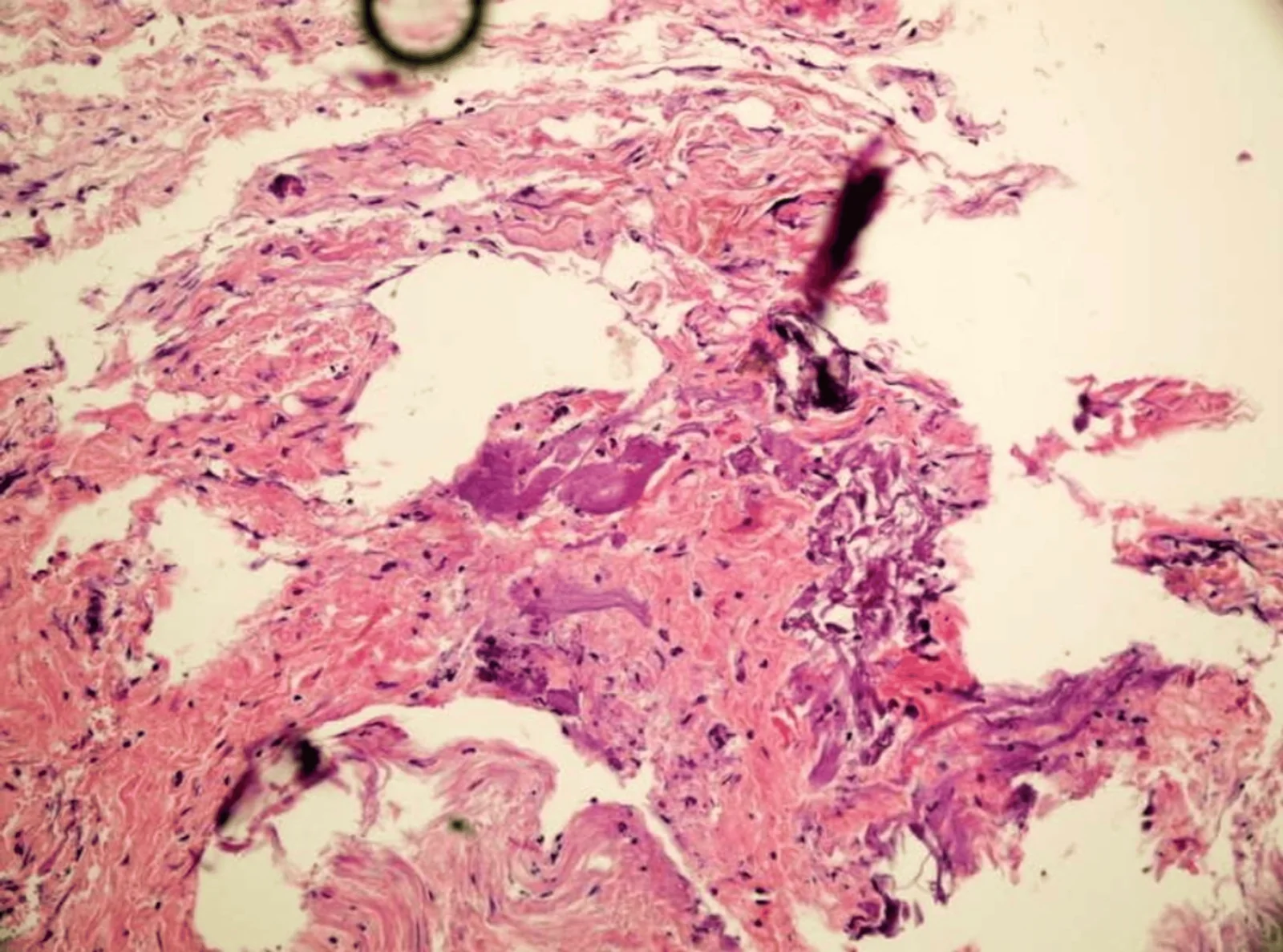
Histopathological Examination of the Excised Encapsulating Membrane (H&E Stain) The specimen demonstrates dense fibrocollagenous tissue with fibroblasts enclosing mature adipose tissue, consistent with sclerosing encapsulating peritonitis. No evidence of malignancy is identified (H&E stain, ×100). H&E: hematoxylin and eosin

Histopathological examination of the excised membrane demonstrated mature adipose tissue surrounded by a thin fibrous capsule, fibroblasts, and thick bundles of collagen fibers, consistent with sclerosing encapsulating peritonitis (Figure 9). After excluding other recognized secondary causes, including abdominal tuberculosis, peritoneal dialysis, chronic peritoneal inflammatory disease, malignancy, and long-term medication use, the patient’s history of four previous Caesarean sections, together with the clinical, intraoperative, and histopathological findings, was considered the most likely predisposing factor, consistent with a diagnosis of secondary type I ACS.

The patient was followed regularly in the outpatient clinic at six months, one year, and two years postoperatively. Throughout the follow-up period, she remained asymptomatic, tolerated a regular diet, and experienced no recurrence of bowel obstruction. At her two-year follow-up visit, she reported complete resolution of her symptoms and expressed satisfaction with the surgical outcome and her overall recovery. 

A chronological summary of the patient’s clinical course is presented in Table 1.

**Table 1:** Clinical Timeline of the Patient’s Presentation, Diagnosis, and Management CT: computed tomography

Time	Clinical event
5 years before presentation	Fourth Caesarean section (most recent abdominal surgery)
Approximately (2-3) months later	Onset of recurrent, self-limiting episodes of colicky abdominal pain and intermittent intestinal obstruction, occurring approximately 3-4 times annually and managed conservatively with bowel rest and intravenous fluid therapy at peripheral clinics
2 years before presentation	Colonoscopy performed to investigate recurrent obstructive symptoms; no abnormalities were identified
1 week before hospitalization	Onset of colicky abdominal pain, progressive abdominal distension, and absolute constipation
Hospital days 1-7	Conservative management with bowel rest and intravenous fluid therapy without clinical improvement
Hospital day 7	Progressive worsening of abdominal pain despite conservative management; plain abdominal radiography demonstrated multiple air-fluid levels consistent with small bowel obstruction; contrast-enhanced CT suggested jejunojejunal intussusception
Hospital day 8	Diagnostic and therapeutic laparoscopy revealed secondary type I abdominal cocoon syndrome; complete laparoscopic excision of the fibrocollagenous membrane was successfully performed
Postoperative days 1-3	Liquid diet resumed on postoperative day 1 and advanced to a soft diet on postoperative day 3
Postoperative day 5	Discharged home in good condition
2 years after surgery	Remained asymptomatic with no recurrence of bowel obstruction, tolerated a regular diet, and expressed satisfaction with the surgical outcome

## Discussion

Abdominal cocoon syndrome (ACS) is an uncommon but clinically significant cause of mechanical small bowel obstruction, typically presenting with recurrent, acute, or chronic episodes of intestinal obstruction caused by compression and kinking of the bowel within a fibrocollagenous encapsulating membrane [5]. Owing to its rarity and nonspecific clinical presentation, patients frequently experience recurrent obstructive symptoms before the diagnosis is established, a finding consistently reported across case series and systematic reviews [6-8]. Despite advances in cross-sectional imaging, preoperative diagnosis remains challenging because the radiological findings are often subtle and variable or overlap with other causes of small bowel obstruction [4]. The present case highlights two important aspects of ACS: the potential for computed tomography (CT) findings to mimic jejunojejunal intussusception and the short-term technical feasibility of laparoscopic management in carefully selected patients.

Several imaging modalities have been described for the evaluation of ACS, including plain abdominal radiography, barium studies, ultrasonography, computed tomography (CT), and, less commonly, magnetic resonance imaging (MRI) [5,8]. Among these, contrast-enhanced CT is considered the most valuable imaging modality for evaluating small bowel obstruction and for preoperative diagnosis and surgical planning, with a reported sensitivity of 73%-95% for high-grade small bowel obstruction; however, its findings in ACS are often nonspecific [4,5,8]. CT findings include centrally clustered or conglomerated small bowel loops encased within a fibrocollagenous membrane, with bowel loop fixation resulting in the characteristic cauliflower-like appearance (“cauliflower sign”), together with peritoneal thickening and enhancement, ascites or loculated fluid collections, reactive adenopathy, and peritoneal or mural calcifications [4,5,9].

In the present case, the predominant CT finding was a target-like configuration, leading to an initial diagnosis of jejunojejunal intussusception. This interpretation was reasonable because the target sign is a well-recognized CT feature of adult intussusception, reflecting the characteristic bowel-within-bowel configuration produced by invagination of one bowel segment into another [10,11]. Furthermore, CT findings suggestive of intussusception with a pathological lead point include bowel obstruction and identification of a lead mass, whereas transient intussusceptions without a lead point are typically not associated with obstructive features [11].

This represents an important diagnostic pitfall, as ACS can mimic intussusception on imaging. Crowding and partial encapsulation of clustered bowel loops may create a pseudo-target configuration on CT imaging. To our knowledge, reports describing this specific radiological mimicry remain extremely limited, underscoring the importance of maintaining a high index of clinical suspicion for ACS in patients presenting with recurrent or unexplained bowel obstruction.

The differential diagnosis of ACS includes congenital peritoneal encapsulation, internal hernia, adhesive small bowel obstruction, and, less commonly, voluminous intussusception, all of which may present with overlapping clinical and radiological features [5,9]. Among these, internal hernia and congenital peritoneal encapsulation are the two most important differential diagnoses [5,8]. Although internal hernias may demonstrate CT findings similar to those of ACS, they lack the characteristic fibrocollagenous encapsulating membrane [8]. In contrast, congenital peritoneal encapsulation is a developmental anomaly characterized by a thin accessory peritoneal sac, whereas ACS is distinguished by a thick fibrocollagenous membrane associated with recurrent intestinal obstruction [8]. Voluminous intussusception may also closely resemble ACS on CT. However, ACS typically demonstrates a sac-like configuration with clustered bowel loops enclosed within a soft tissue fibrocollagenous membrane, whereas voluminous intussusception appears as a large elongated bowel loop extending along the longitudinal axis of the intestine. In addition, the soft tissue rim in intussusception represents the telescoped bowel segment, which is characterized by greater mural thickness and identifiable mucosal folds, findings that are absent in ACS [12]. Awareness of these entities may facilitate earlier recognition of ACS and help avoid diagnostic delay [5].

Careful retrospective postoperative review of the CT images revealed previously overlooked central clustering of vertically oriented small bowel loops enclosed within a thin enhancing fibrocollagenous membrane. A small amount of intraperitoneal fluid and subtle omental thickening were also present, findings characteristic of ACS. However, these findings were initially obscured by the more prominent pseudo-target appearance. Recognition of ACS on CT requires careful interpretation of imaging findings in conjunction with the patient’s clinical presentation, as characteristic features may be overlooked or attributed to more common causes of bowel obstruction. Accordingly, radiologist awareness and meticulous image assessment are essential to recognize these characteristic findings and avoid preoperative misdiagnosis.

A similar diagnostic challenge has been reported in the opposite direction by Li and Zhang, who described extensive midgut intussusception closely resembling ACS because of overlapping imaging features [12]. To the best of our knowledge, no previous report has described ACS initially interpreted as jejunojejunal intussusception on CT.

ACS can be classified according to the extent of bowel involvement into three anatomical types: type I, in which only a segment of the small bowel is encapsulated; type II, characterized by complete encapsulation of the entire small bowel; and type III, in which the fibrocollagenous membrane extends to involve other intra-abdominal organs [13,14]. Based on the intraoperative findings, our patient had type I disease.

Because ACS is a rare disease, evidence-based management guidelines are lacking. Nevertheless, published reviews consistently recommend conservative treatment for carefully selected patients with mild or intermittent obstructive symptoms, whereas surgery remains the definitive treatment for patients with persistent or complete bowel obstruction [5,8]. Conservative management includes bowel rest, nasogastric decompression, fluid and electrolyte replacement, and nutritional support [5,8]. However, because conservative therapy does not address the underlying fibrocollagenous encapsulating membrane, recurrent obstructive episodes are common, and definitive treatment usually requires surgical intervention [5,15]. Adjunctive medical therapy has been reported in selected patients who fail conservative treatment. Agents including corticosteroids, tamoxifen, colchicine, azathioprine, and mycophenolate mofetil have been used; however, the available evidence remains limited and is derived predominantly from patients with secondary SEP, particularly those receiving peritoneal dialysis, rather than idiopathic or postoperative ACS [5,8,15].

Surgical exploration is recommended in patients with complete bowel obstruction, bowel ischemia or perforation, or failure of conservative management [8,13,15]. The primary surgical objectives are complete excision of the fibrocollagenous membrane and meticulous adhesiolysis to release the entrapped bowel loops while preserving bowel viability [5,14]. Intestinal resection should be avoided whenever possible and reserved only for patients with nonviable, gangrenous, or perforated bowel because unnecessary bowel resection increases postoperative morbidity and mortality [5,13,14].

Although laparotomy has traditionally been regarded as the standard surgical approach, successful laparoscopic management has also been reported in carefully selected patients [13,14,16]. Laparoscopy serves as both a diagnostic and therapeutic modality but remains technically demanding because of dense adhesions and the risk of iatrogenic bowel injury during trocar insertion and adhesiolysis. Therefore, it should be performed by surgeons experienced in advanced minimally invasive surgery [5,8,13,16]. In our patient, laparoscopy enabled definitive diagnosis and complete excision of the encapsulating membrane with meticulous adhesiolysis without bowel resection or conversion to laparotomy. Nevertheless, this single case demonstrates the short-term feasibility of laparoscopic management in a carefully selected patient. A comparison of selective representative studies describing the preoperative imaging findings and surgical management of abdominal cocoon syndrome is summarized in Table 2.

**Table 2:** Comparison of Selected Representative Studies on Preoperative CT Findings and Management of ACS ACS: abdominal cocoon syndrome, CT: computed tomography

References	Study design	Characteristic CT findings	Management approach
Makam et al. (2008) [16]	Case report	Diagnosed intraoperatively as CT was not performed; plain abdominal radiography and ultrasonography suggested small bowel obstruction	Diagnostic and therapeutic laparoscopy with complete membrane excision and adhesiolysis
Wei et al. (2009) [6]	Case series	Clustered small bowel loops encased within a thick fibrous membrane (“cocoon”)	Laparotomy with membrane excision and adhesiolysis; intestinal resection was reserved strictly for nonviable segments
Ertem et al. (2011) [17]	Case report	Clustered small bowel loops enclosed within a sac-like structure with proximal jejunal dilatation	Successful laparoscopic adhesiolysis and complete membrane excision without bowel resection
Aziz et al. (2021) [18]	Case report	Clumped small bowel loops surrounded by a thin encapsulating membrane with a transition point at the ileocecal junction	Successful laparoscopic membrane excision and adhesiolysis
Rupani et al. (2026) [19]	Case report	Clustered distal ileal loops surrounded by an enhancing membrane without a definite transition point	Successful diagnostic and therapeutic three-port laparoscopy with complete membrane excision and adhesiolysis

The prognosis of ACS is generally excellent following complete surgical excision of the fibrocollagenous membrane and meticulous adhesiolysis, with most patients remaining symptom-free, and recurrence appears uncommon after complete membrane excision. Despite these favorable outcomes, long-term follow-up is advisable to detect recurrent bowel obstruction or postoperative adhesive complications [8,14,15]. In the present case, the patient remained asymptomatic without recurrence during two years of follow-up, consistent with the favorable outcomes reported in the literature.

In this context, clinical correlation remains essential when radiological findings are inconclusive or misleading. This case expands the spectrum of radiological manifestations of ACS by demonstrating that it may closely mimic intussusception on CT. It also supports the role of laparoscopy as a feasible diagnostic and therapeutic option in carefully selected patients when performed by experienced surgeons, offering several potential advantages, including reduced postoperative pain and shorter recovery. However, further studies and larger case series are needed to better define the role of minimally invasive surgery in the management of ACS.

This report has several limitations. First, the retrospective reinterpretation of the CT images after the intraoperative diagnosis may have introduced confirmation bias in recognizing imaging features suggestive of abdominal cocoon syndrome. Second, this report describes a single patient; therefore, the findings should be interpreted cautiously and cannot establish the safety, generalizability, superiority, or long-term effectiveness of laparoscopic management despite the favorable clinical outcome observed during two years of follow-up.

## Conclusions

ACS should be included in the differential diagnosis of patients presenting with recurrent or unexplained small bowel obstruction. CT findings may be misleading and mimic more common conditions such as intussusception, thereby underscoring the importance of clinical vigilance. This case contributes to the limited but growing literature supporting laparoscopic excision as a feasible diagnostic and short-term therapeutic option for selected patients with secondary type I ACS in experienced hands. However, these findings should be interpreted with caution given the inherent limitations of a single case report. Larger multicenter case series are needed to better define the optimal diagnostic and surgical management strategies for ACS.
